# General Stress Responses in the Honey Bee 

**DOI:** 10.3390/insects3041271

**Published:** 2012-12-11

**Authors:** Naïla Even, Jean-Marc Devaud, Andrew B. Barron

**Affiliations:** 1 Department of Biological sciences, Macquarie University, NSW 2122, Australia; E-Mail: andrew.barron@mq.edu.au; 2 Centre de Recherches sur la Cognition Animale, Université de Toulouse, UPS, 118 route de Narbonne F-31062 Toulouse Cedex 9, France; E-Mail: jean-marc.devaud@univ-tlse3.fr; 3 CNRS, Centre de Recherches sur la Cognition Animale, 118 route de Narbonne F-31062 Toulouse Cedex 9, France

**Keywords:** honey bee, *Apis mellifera*, stress, *corpora cardiaca*, dopamine, octopamine, allatostatin, corazonin, adipokinetic hormone, diuretic hormone

## Abstract

The biological concept of stress originated in mammals, where a “General Adaptation Syndrome” describes a set of common integrated physiological responses to diverse noxious agents. Physiological mechanisms of stress in mammals have been extensively investigated through diverse behavioral and physiological studies. One of the main elements of the stress response pathway is the endocrine hypothalamo-pituitary-adrenal (HPA) axis, which underlies the “fight-or-flight” response via a hormonal cascade of catecholamines and corticoid hormones. Physiological responses to stress have been studied more recently in insects: they involve biogenic amines (octopamine, dopamine), neuropeptides (allatostatin, corazonin) and metabolic hormones (adipokinetic hormone, diuretic hormone). Here, we review elements of the physiological stress response that are or may be specific to honey bees, given the economical and ecological impact of this species. This review proposes a hypothetical integrated honey bee stress pathway somewhat analogous to the mammalian HPA, involving the brain and, particularly, the neurohemal organ *corpora cardiaca* and peripheral targets, including energy storage organs (fat body and crop). We discuss how this system can organize rapid coordinated changes in metabolic activity and arousal, in response to adverse environmental stimuli. We highlight physiological elements of the general stress responses that are specific to honey bees, and the areas in which we lack information to stimulate more research into how this fascinating and vital insect responds to stress.

## 1. Introduction

### 1.1. Concept of Stress

The term stress originated in physics to describe pressure and deformation in a system, but it has been adopted into a biological context through the work of Hans Selye [1,2,3]. He recognized in mammals, as a “general adaptation syndrome,” a similar suite of coordinated reactions to diverse noxious stimuli or “agents” [4]. Selye’s concept was at first criticized by physiologists as vague and immeasurable, but he subsequently clarified his concept defining several stress response elements, principally the hypothalamo-pituitary-adrenal (HPA) axis system. Stress is now recognized as a valid physiological concept, which allows organisms to respond to adverse environmental pressures [5]. Most studies use the word “stress” to describe negative treatments applied to organisms in an experiment, such as nutritional, heat or oxidative stress. Here, the triggering stimuli will be called “stressors” while “stress” will be considered as the response syndrome to any aversive or harmful treatment in a specific system. This understanding can be applied to different levels of organization: molecular, cellular, histological, physiological, even ecological or social, but this review will focus on physiological processes involved in an integrated response at the level of the organism. Also, the definition of stress should take into account the duration and intensity of the stressors involved, thereby the distinction between acute or chronic stress responses. Here, we review the putative elements participating in a physiological stress response, and propose an integrated model of the honey bee stress response. Although the model is based to a degree on the stress literature known from other insects [6,7,8,9], our intention is to build (as far as possible) a model that is honey bee specific. In doing so, we identify what is known about this particular species, and what may be assumed from our knowledge of other insects. Consistently, we wish to highlight the gaps in our existing knowledge to propose directions for future stress research.

### 1.2. Why Study Stress in Honey Bees?

The concept of stress is useful in understanding the physiological and behavioral responses of honey bees to harmful situations. This research is timely since, over the last years, beekeepers from different geographic areas have reported a marked increase in honey bee colony failure rates and in the number of stressors affecting bees, including diseases, parasites, pesticides and poor nutrition [10,11,12]. The syndrome termed “colony collapse disorder” seems to be the result of an accumulation of stressors chronically weakening honey bee colonies [13,14]. As honey bees are the most important insects in agriculture for both pollination of diverse crops and honey production, the recent decline in their populations brings an urgent need to know more about the stress response systems of this ecologically and economically important insect.

In addition, the honey bee is an ideal insect model to understand the evolution of sociality. A key feature of honey bees is their high level of social organization and their well-developed system of division of labor among workers [15]. Honey bees exhibit age polyethism; young workers perform in-hive tasks (e.g., taking care of the brood), then become guards patrolling the entrance of the hive and later become foragers. Studying differences in stress responses across behavioral castes might help elucidate how a defined division of labor has evolved.

### 1.3. How Does an Organism React to Stress?

**Figure 1:**
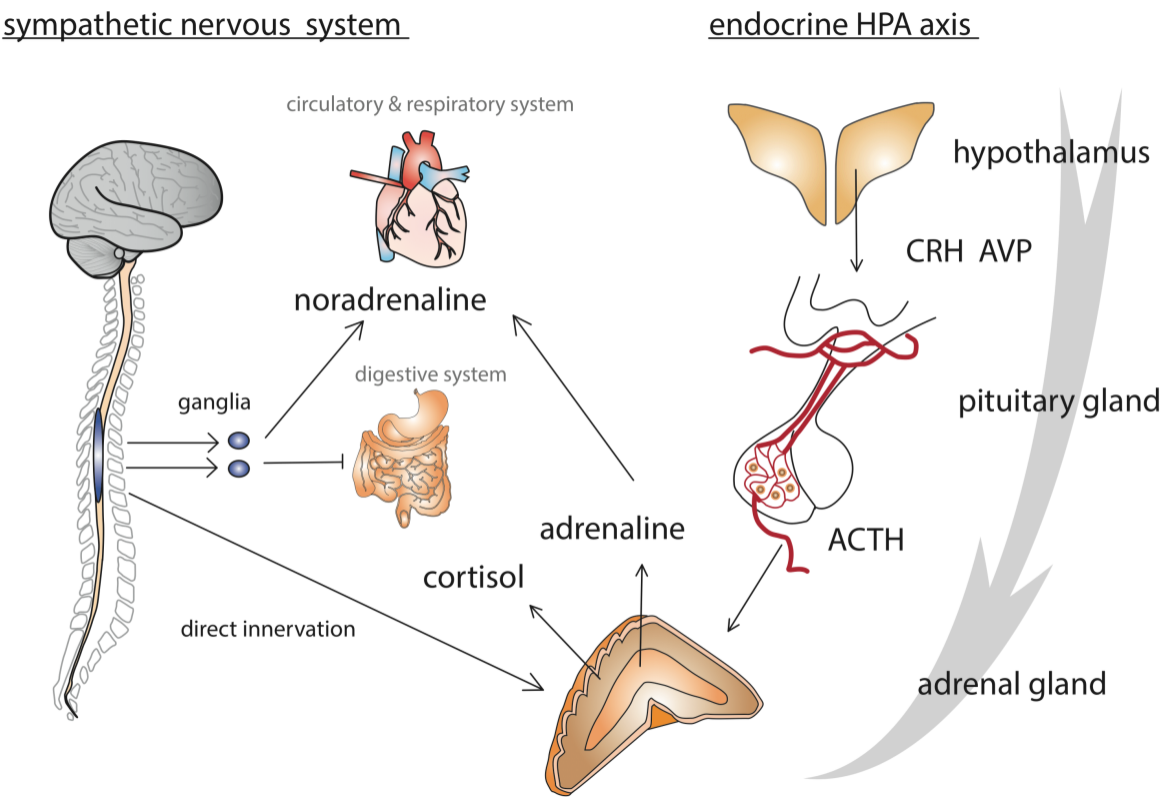
Human stress response pathways operating through the autonomic nervous system and the endocrine system. This diagram illustrates how neural and hormonal signals interact and complement each other through the regulatory action of the hypothalamo-pituitary-adrenal axis (HPA axis). ACTH: adreno-corticotropin hormone. AVP: arginin/vasopressin. CRH: cortico-releasing hormone. Adapted with permission from Macmillan Publishers Ltd.: Nature Reviews Neuroscience [25], copyright 2009.

There are three stages to an organism’s acute stress response: it first detects the stressor with sensory organs, then responds to it by defense or escape. Finally, if the stressor cannot be avoided and is sustained, the organism enters a state of exhaustion [1]. Following detection of a stressor, mammalian physiological responses are coordinated by neural activity within the autonomic nervous system and the HPA axis (Figure 1). The first immediate response is an activation of sympathetic neurons, which stimulate the adrenal medulla to release adrenaline and noradrenalin into the blood. These two catecholamines increase heart rate and vasoconstriction. In parallel, the paraventricular nucleus (PVN) neurons in the hypothalamus release corticotrophin-releasing hormone (CRH) and arginine/vasopressin (AVP), which are conveyed to the nearby anterior pituitary gland via the blood stream. In response, the pituitary secretes adrenocorticotropic hormone (ACTH), which acts on the adrenal cortex to release glucocorticoids (such as cortisol) causing mobilization of energy reserves (*i.e*., glucogenolysis in the liver) [16]. Glucocorticoids also potentiate catecholamine release from the adrenal medulla. In parallel, adrenaline activates a release of glucagon by the pancreas to further increase the catabolism of glycogen in the liver and raise glucose concentration in the blood. Other hormones such as cytokines or endogenous opioids may also be produced and/or released, depending on the nature, duration and intensity of the stressor, to act in diverse ways to limit the degree of tissue damage. Therefore, it is interesting to note that, additionally to the general stress response pathways described previously, certain stress responses can vary depending on the type and duration of the stressor.

Under chronic stress, the immune system, metabolic pathways and cognitive processes in the organism gradually weaken until exhaustion and failure are reached [1,17]. For example, repetitive HPA activation resulting in an excess of glucocorticoids in the blood can lead to metabolic diseases such as diabetes [2,18].

Cellular stress responses described in various models (bacteria, yeast, worms and flies) include the increased production or activation of antioxidant proteins and heat shock proteins (HSP) when facing high metabolic load or environmental stressors [19,20]. Such proteins may be called “stress proteins” [21] and used as cellular stress biomarkers [22,23]. These factors are induced by a variety of stressors such as extreme temperatures, elevated ion concentrations or toxic substances, all usually resulting in excessive amounts of denatured proteins [24]. Their actions are principally intracellular and hence we do not focus on them in this review that considers instead more integrated elements of a general stress response.

## 2. Model of the Honey Bee Stress Response

### 2.1. How Has Stress Been Assessed in Honey Bees?

**Figure 2:**
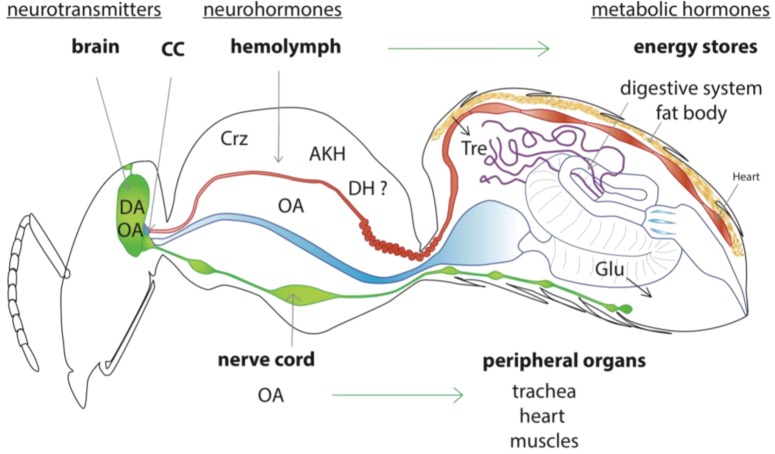
Hypothesized model of the general stress system in the honey bee. The brain biogenic amines (OA) and dopamine (DA), acting as neurotransmitters or neuromodulators, would increase arousal, cognitive processes and sensitivity to various stimuli. Then, neurosecretory cells of the *corpora cardiaca* (CC), would release metabolically active hormones into the hemolymph. These may include corazonin (Crz), adipokinetic hormone (AKH), and possibly diuretic hormone-I (DH). This cocktail of hormones mobilize energy from the midgut and the fat body (see detail in Figure 3). Activation of the octopaminergic DUM (Dorsal Unpaired Median) neurons of segmental ganglia of the ventral nerve cord would stimulate activity of skeletal and visceral muscle. Metabolic hormones like allatostatinA, tachykinin-related and insulin-related peptides can be released from peripheral neurosecretory cells, where they can modulate gut motility, and may also contribute to regulate the release of Crz, AKH and DH from the CC.

Multiple aspects of stress responses have been used to evaluate stress in honey bees, including behavioral, physiological or cellular stress responses. The many parameters used are listed in Table 1. Behavioral stress responses, usually immediate responses, characterized early on by Cannon [26] as the “fight-of-flight” responses, are easy to observe in the honey bee. For example, extension of the sting (or stinging of a target) has been used to evaluate sensitivity to stressors, and is widely considered as indicative of stress in honey bees, as well as an aggressive response. Physiological measures of stress responses in honey bees include hormonal titers and neurotransmitter levels—these parameters have been integrated into our model (see Table 1 and Table 2, Section 2.2 and Section 3 and Figure 2). Honeybee stress studies usually use acute stressors but the nature and duration of the stressors could sometimes be qualified as chronic (Table 1). Cellular stress responses have also been used in honey bees [27,28,29,30,31,32], and Duell *et al.* [33] even suggest some cellular stress biomarkers as elements for a diagnostic of general stress in honey bees. Also, since exhaustion is the final stage of chronic stress response described by Selye [1], survival rate has been used to assess the degree of stress.

**Table 1:** Stress response measures used in honey bees. This inventory illustrates the broad diversity of methods used to evaluate stress in honey bees. The table is divided into four parts, depending on the measure used: behavioral, physiological, cellular and survival. In the absence of an objective distinction between chronic and acute stressors, here we qualify stressors ingested or applied continuously during at least four hours as chronic (C); otherwise they are qualified as acute (A).

stress response measure	stressor	acute/chronic	variable	references
physiological responses				
juvenile hormone (RIA)	cold anesthesia, caging	A	task specialization, duration after treatment	Lin *et al.*, 2004 [34]
brain biogenic amines (HPLC)	spinning, caging, chilling, CO_2_	A	spinning speed, duration of stressor	Chen *et al.*, 2008 [35]
brain biogenic amines (HPLC)	leg pinch	A	duration of stressor, age, season, patriline	Harris and Woodring, 1992 [36]
cellular stress responses				
HSP 70 (Elisa)	capture, transport, chilling, harnessing	A/C	ethanol concentration, duration of harness	Hranitz *et al.*, 2010 [31]
HSP70 (western)	heat	A	duration of stressor, age body part	Elekonich, 2009 [28]
hsp70, hsc70 (q PCR)				
CRH-BP (qPCR)	cold, heat, UV light	A	intensity of stressor, caste, development stage, body part	Liu *et al.*, 2011 [37]
behavioral response				
stinging response	electric shock	A	patriline	Lenoir *et al.*, 2006 [38]
stinging response (delay)	electric shock	A	genotype, housing conditions,	Uribe-Rubio *et al.*, 2008 [39]
			task specialization	
sting extension	electric shock	A	genotype, exposure to alarm pheromone	Balderrama *et al.*, 1987, 2002 Roussel *et al.*, 2009 [40,41,42]
			task specialization	
sting extension	electric shock	A	morphine and opioid peptides treatment	Núñez *et al.*, 1983, 1997 [43,44]
proboscis extension	soil-borne pollutants	C	treatment concentration	Hladun *et al.*, 2012 [45]
survival				
survival	hyperoxia	C	learning performance	Amdam *et al.*, 2010 [46]
survival	paraquat injection	C	vitellogenin level, reproductive castes	Seehuus *et al.*, 2006; Corona *et al.*, 2007 [47,48]
	(oxidative stressor), hyperoxia			

**Table 2:** Main elements of the general stress response in the honey bee. The table gives the physiological role(s), known or hypothesized (as indicated by a question mark) for each element, following a classification based on chemical identity.

chemicals	abbreviation	nature	stress-related action	references
biogenic amines				
octopamine	OA	neurotransmitter	enhances arousal, increases heart rate, modulates muscle activity	Corbet, 1991; Farooqui, 2012,
				Papaefthimiou and Theophilidis, 2011,
				Pflüger *et al.*, 2004 [49,50,51,52]
		neurohormone		
dopamine	DA	neurotransmitter	modulates arousal	Mustard *et al.*, 2010 [53]
peptides				
adipokinetic hormone	AKH	hormone	mobilize energy in the fat body	Kodrik, 2008 [54]
cortico releasing hormone-binding protein	CRH-BP	chaperone?	potentiates or inhibits hormonal release ?	Liu *et al.*, 2011 [37]
diuretic hormone-I	DH-I	hormone	stimulates diuresis induced by crop draining into hindgut after energy mobilization.	Coast *et al.*, 2002 [55]
corazonin	Crz	neurohormone	activates metabolism?	Veenstra, 2009 [56]
allatostatin-A	AST-A	neurohormone	activates gut contraction ? inhibits corazonin neurosecretion ?	Veenstra, 2009 [56]
insulin-like peptide	ILP	?	regulates energy stores ?	Corona *et al.*, 2007 [48]
proteins				
heat shock proteins	HSP70	chaperone	protects cells against oxidative stress and excess protein misfolding	Hranitz *et al.*, 2010, Elekonich, 2009 [28,31]
ERK2	ERK2	?	protects cells against damage ?	Li *et al.*, 2012 [30]
vitellogenin	Vg	antioxidant	protects cells against damage	Seehuus *et al.*, 2006 [47]

Based on the data available for honey bees (Table 1) and other insect species we have tried to synthesize a model of a general stress response pathway specific to the honey bee. It should be kept in mind that many proteins or genes of unknown function may be affected by stressors; we will only focus on a few of them, for which sequence homologies and/or functional data suggest a potential role in a physiological stress response.

### 2.2. Model of the Honey Bee Stress Response

When faced with a stressor such as predation, robbing or adverse climatic conditions, a honey bee will need to increase her mobility and mobilize energy reserves to cope with the sudden increase of metabolic demand. We suggest here that this rapid change in physiology is achieved by a coordinated endocrine and neuroendocrine response (Figure 2).

**Figure 3:**
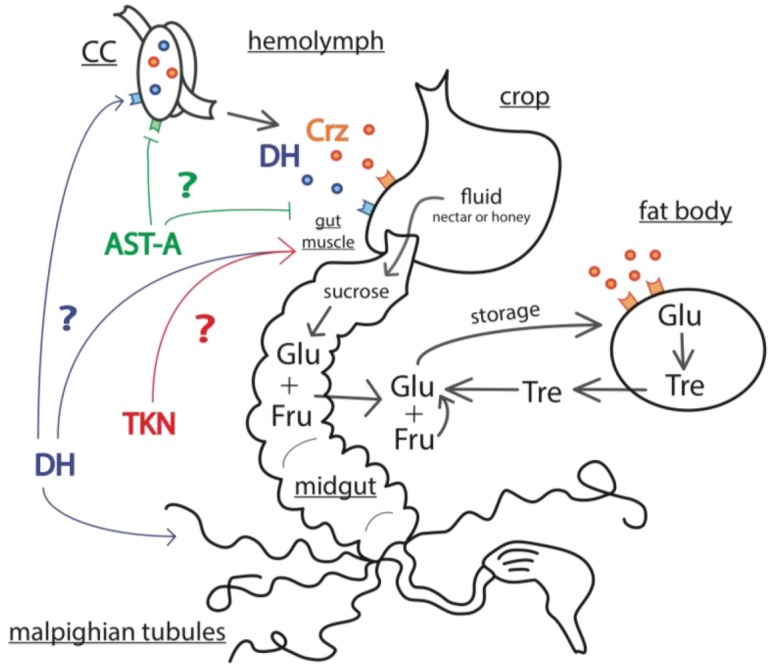
Hypothetical model of energy mobilization in honey bee. Glucose (Glu) and trehalose (Tre) are the main sources of energy in the hemolymph. Trehalose is stored in the fat body and, when necessary, is released into the hemolymph to be metabolized into glucose. Another source of hemolymph glucose is sucrose from nectar contained in the crop. If hemal carbohydrate levels drop, an influx of nectar is passed from the crop to the midgut via muscle contractions. In the midgut, sucrose is metabolized into fructose (Fru) and glucose, which are then transported to the hemolymph. The passage of nutrients from the crop to the midgut is allowed by contraction of the gut muscle, also named the proventriculus. During normal metabolic demands this influx from the crop depends on the carbohydrate concentration in the hemolymph [61,62].In energy-demanding situations, this process might be boosted by tachykinin-related peptides (TRPs), diuretic hormone-I (DH-I), corazonin (Crz) and adipokinetic hormone (AKH) while an inhibitory effect from allatostatin A (AST-A) secreted from the midgut would be relieved. DH-I may also exert feedback on corazonin-secreting cells of the *corpora cardiaca* (CC).

As soon as the stressor is detected via appropriate receptors (e.g., olfactory, mechanosensory or visual), our model proposes that there will be release of octopamine and dopamine within the brain, thus increasing arousal [49] (see Section 3.1). Other signals like cortico-releasing hormone-binding protein (CRH-BP) might also participate in the brain stress response [37]. Octopamine is also released into the hemolymph [57] from neurohemal cells [58] to act on many organs and coordinate a physiological response to the stressor (see Section 3.2.1). Peripheral octopamine increases heart rate, and may modulate ventilation and stimulate mobilization from muscles [51,59]. The activation of the neurosecretory cells of the *corpora cardiaca* (CC), the major brain neurosecretory organ, stimulates the release of several neurohormones: adipokinetic hormones (AKH) and possibly corazonin, into the hemolymph [6,54,56] to mobilize energy from body stores (see Section 3.2). Finally, we suggest here (from honey bee physiology studies) that hormonal factors including AKH and candidates like allatostatin-A (AST-A), diuretic hormones (DHs) and tachykinins [56], could reinforce the liberation of trehalose and glucose from the fat body, but also from the main energy store of the honey bee—the crop [56,60,61] (see Section 3.3 and Figure 3).

## 3. Molecular Signals of the Honey Bee Stress Response

### 3.1. Stress Indicators within the Brain

#### 3.1.1. Biogenic Amines

The role of catecholamines as hormones and neuromodulators in the acute stress response is extremely well conserved and well documented in vertebrates [63]. In insects, the biogenic amines octopamine and dopamine are also involved in responses to stressors [8,64,65]. Their respective receptors are phylogenetically related to adrenergic and dopaminergic receptors [50,66,67], showing a strong conservation of both structure and function through more than 500 million years of evolution. These amines regulate many aspects of insect physiology and behavior [50,67], but principally have been shown to increase arousal state and motor activity in several insect species [49,68]. In *Drosophila*, dopamine modulates sleep and locomotion, thus paralleling the functions of dopamine in mammalian circadian rhythms and arousal state [69,70]. Similarly, octopaminergic neurons from the *pars intercerebralis* regulate the sleep:wake cycle [71] (“endogenous arousal”) and octopamine signaling has been implicated in arousal increase in response to environmental stressors (“exogenous arousal”) [49]. Both forms of arousal are inversely regulated by dopamine, which exerts an inhibitory control on stressor-induced locomotor hyperactivity [72]. In the honey bee, there is evidence that dopamine and octopamine modulate motor activity [53,73]. In many insects, including honey bees, octopamine treatments have been shown to increase sensitivity to sensory inputs [68,74,75,76,77]. Moreover, two studies have shown that exposure to physical stressors modifies brain levels of octopamine and dopamine in honey bees [35,36].

Both octopamine and dopamine also modulate learning of a stressful event, particularly dopamine [78,79,80]. In this regard, the functions of these biogenic amines parallel those of catecholamines (adrenaline and noradrenalin) in mammals, which modulate not only the initial neurohormonal cascade of the stress response, but also the learning of a stressful event [81]. Therefore, both in mammals and invertebrates, signaling through biogenic amines mediates both the initial stress response and the capacity to learn about the stressors triggering the response, thus potentially modulating behavioral and physiological reactions upon further exposure to these stressors.

#### 3.1.2. Cortico-Releasing Hormone-Binding Protein (CRH-BP) and Its Putative Diuretic Hormone Ligand (DH-I)

Cortico-releasing hormone (CRH), also called cortico-releasing factor (CRF), is a crucial signaling element within the vertebrate HPA axis [82]. Its action is negatively regulated by the CRH-binding protein (CRH-BP) [83] The CRH receptor and CRH-BP are strikingly conserved both structurally and functionally throughout vertebrates as hormonal regulatory elements of the stress response [84,85,86]. CRH-BP even shows a degree of conservation in honey bees [85]. The predicted *Apis mellifera* CRH-BP shares only 25%–29% identity with the vertebrate CRH-BP, but the sequence comparison reveals that amino acids potentially crucial for the 3D structure (cysteines forming bisulfure bridges) [85] and for ligand binding are conserved. Interestingly, its homolog in the Asian honey bee, *Apis cerana* (*Acc*CRH-BP), is expressed as the transcriptional level in the brain [37], and upregulated following application of various acute stressors such as UV light, heat or cold [37]. This increase by diverse stressors strongly suggests a signaling role in general stress pathways, even though the role of CRH-BP in insects (chaperone protein or link with the hormonal cascade) needs to be explored [83]. 

Despite this apparent conservation of CRH-BP in insects, no obvious homolog of CRH has been found yet, but precursor peptides of the vertebrate CRH family display similarities with the insect diuretic hormone-I (DH-I, also named DH31 in *Drosophila*) [84,85,87] which has been suggested to be a good candidate ligand for CRH-BP [85]. Still, a clear link between DH-I and the stress response is lacking, but we note that the regulation of water balance via DH-I action on the excretory system [55] could be essential to mobilize energy sources from the honey bee crop. Since DH-I is detected in the CC [88], it might well be part of a coordinated neuroendocrine cascade preparing the honey bee for rapid energy mobilization in energy-demanding situations (see Section 3.3 and Figure 3). Therefore, we think that DH-I and CHR-BP are good candidates as putative elements of the stress response whose action would be worth considering in the future.

### 3.2. Coordinated Peripheral Stress Responses

In the periphery, the immediate physiological stress response might be coordinated by nerve signals allowing a very fast reaction, but (as in vertebrates) neuroendocrine systems seem to also play a major role in honey bees and other insects. Important components are neurosecretory cells of the CC, which integrate neuronal signals and may trigger broad effects in a variety of target cells through endocrine signals in the hemolymph [89]. Like the vertebrate pituitary gland, the insect CC houses many neuroendocrine cells that play a central role in the regulation of diverse metabolic functions [90].

#### 3.2.1. Octopamine

Additionally to its role as a neurotransmitter and a neuromodulator in the brain, octopamine also acts in periphery, mainly as an endocrine signal. Increases of octopamine level in the hemolymph have been measured in energetically demanding, “fight-or-flight” situations [8,50,57,59,67]. A large literature from locusts, cockroaches, flies and moths demonstrates that many insect organs are sensitive to octopamine, including flight and visceral muscles [91,92,93], reproductive organs [94,95,96,97,98], heart [99,100,101,102], air bags [103], sense organs [104], metabolic tissues such as the fat body [105,106,107,108,109] and malpighian tubules [110,111]. These two latter organs have key roles in energy mobilization in honey bees (see Section 3.3 and Figure 3). Hence, octopamine is in the position to trigger broad and coordinated physiological changes such as the ones expected in a general stress response [112,113]. Several authors have proposed it to be the major stress hormone in insects, including honey bees [8,50,59,67,114], but its specific action on each cell population remains to be clarified in detail [50,67].

In response to threats, octopamine primes flight and leg muscles in locusts [52,115,116]. A similar action in honey bees has not been demonstrated yet, but would be consistent with its positive action on locomotor (specifically flight) activity [73]. In parallel, octopamine seems to also be a cardiostimulant and an activator of the respiratory system to increase oxygen supply to muscles. Modulation of the respiratory system by octopamine is not well understood in insects, but octopamine can stimulate respiratory activity through increasing the hemolymph circulation in the Dobson fly, *Corydalus cornutus*, and the locust *Schistocerca americana* [68,117]. Octopamine was also shown to stimulate respiratory neurons in *Locusta migratoria* [118]. Evidence of similar respiratory effects of octopamine in honey bees is still lacking, and thus more research is needed in this area. Recently, Papaefthimiou and Theophilidis [51] have shown *in vitro* a biphasic effect of octopamine on heart activity in honey bees. A high concentration of octopamine increases the contraction frequency of the heart, but a low concentration has the opposite effect. The authors argue that this double action may indicate the presence of different types of octopamine receptors on the heart, but another explanation may be due to the participation of diverse signaling pathways depending on OA concentration, since receptor activation can trigger different intracellular signals for different OA concentrations, at least *in vitro* [119].

To perform all these diverse functions, octopamine very likely acts both as a neurotransmitter and as a neurohormone [64]. While the distribution of octopaminergic neurons has been described in detail in the honey bee brain and subesophageal ganglion [58,120,121,122] very little is known about its distribution in the nerve cord and motor nerves. This state of knowledge contrasts heavily with the well-characterized network of extensive efferent unpaired octopaminergic neurons in locusts and cockroaches [123,124,125,126]. Thus, data from these species suggest that such neurons might act similarly to the sympathetic vertebrate system by releasing octopamine from varicosities directly near the organs (glands, peripheral flying or leg muscles) [127,128]. In the honey bee, octopamine-like immunoreactivity in varicose structures of CC suggests a possible (neuro)endocrine source of octopamine [58]. Additionally, our model assumes that a network of peripheral octopaminergic neurons exists in honey bees as in locusts, but information on this is currently lacking. It will be important to confirm the presence of octopaminergic neurohemal structures on the surface of peripheral nerves similar to those described in locusts, which are only inferred in honey bees for now, based on comparison with various insects [50,123].

Octopamine is released in energy demanding “fight-or-flight” situations to increase the honey bees’ state of general arousal and we can therefore consider it as a stress hormone in insects [8,50,114]. Interestingly, octopaminergic neurosecretory cells innervate the honey bee CC [58,120], thus suggesting that octopamine could also regulate the release of several neuropeptides from this structure (including the stress candidates discussed hereafter).

#### 3.2.2. Corazonin

The cardioacceleratory function of this 11-aminoacid neuropeptide was first described in 1989 by Veenstra in *Periplaneta americana* [129]. Now we know that this effect is probably restricted to cockroaches only, while corazonin has been shown to have diverse effects in other insects such as silkworms, locusts, flies, and moths. In locusts (both *Locusta migratoria* and *Schistocerca gregaria*), corazonin is involved in the induction of the gregarious phase [130], and in ecdysis in the moth *Manduca sexta* [131]. Also, a metabolic function of corazonin as a nutritional stress hormonal signal has been recently suggested by Veenstra [56], based on the localization of the peptide precursor and its receptor in *Drosophila*. Corazonin is produced by neurosecretory cells projecting into the CC in many insects, including *Drosophila*, locusts and honey bees [132,133]. In *Drosophila*, corazonin receptors have been found in the heart, fat body, salivary glands and gut [134]. Additionally, in *Drosophila*, corazonin neurons express diuretic hormone receptors and an AST-A receptor [135]. This has led Veenstra to suggest a model in which corazonin is released in response to peripheral feedback from the gut in a hunger state, and acts to mobilize energy (see Section 3.3 and Figure 3). Phylogenetic analyses support an ancestral hormonal role for corazonin in regulating metabolic functions, more specifically because corazonin receptors belong to the ancient GnRH-AKH receptor family [56,136,137]. Based on these recent findings on the role of corazonin in insects we propose, following others [6], that it may have an important hormonal role in the honey bee acute and chronic stress response, although this hypothesis has yet to be directly tested.

#### 3.2.3. Allatostatins

Allatostatins (ASTs) are insect neuropeptidic hormones first identified as regulators of growth during development, on the basis of their ability to reduce juvenile hormone (JH) release by the CC [138]. However, there is growing evidence that not all members of the AST family play this biological function [138,139,140] Among the three major AST types (A, B and C), only A and C are present in honey bees [139,140]. In addition, honey bees (as along with some other insect species) have two closely related C-type peptides, AST-C and AST-CC [141].

ASTs are important regulators of food intake and/or digestive functions in several insect species [142,143,144,145,146], but this might be part of a much broader spectrum of inhibitory functions [141]. They are present in the midgut of several species as well as in the CC [143,147,148], and are known to release neuroendocrine signals regulating energy supply from the digestive tract (see below and Figure 3). Hemal AST-A has been suggested to modulate CC function [56]: low food content in the gut reduces circulating AST-A released by midgut secretory cells; this in turn relieves inhibition of CC neuroendocine cells containing corazonin and diuretic hormones. This postulated role in response to nutritional stress has been recently challenged by recent work in *Drosophila*, showing that genetic manipulations of AST-A alter feeding behavior without apparent consequences on energy reserves or metabolism [149]. Thus, whether an AST-mediated nutritional feedback loop exists remains an open question. It is worth mentioning that at least the AST-A type may be expressed in the honey bee CC [150] (but see [88]), which places it in a position of possibly participating in energy mobilization control, in particular under conditions of chronic and acute nutritional stress. 

#### 3.2.4. Adipokinetic Hormone (AKH)

AKH is perhaps the most important metabolic regulator described in insects [109]. This octapeptide is synthesized in the CC and released into the hemolymph to increase catabolism in the fat body, ultimately leading to increased circulating trehalose levels [7,151] (see detail in the following section), similarly to the action of glucagon in vertebrates [152,153,154]. In cockroaches, AKH stimulate spiking from peripheral octopaminergic neurons and locomotion [155]. Interestingly, the demonstration that octopamine mediates AKH release into the hemolymph in the locust CC [156,157,158] provides further evidence of a precise interplay between arousal and hunger. In addition, recent papers support a role for AKH in a general stress response in various insects [54,159,160]. Insecticide treatment inducing oxidative stress leads to increased hemolymph titers in the locust *Schistocerca gregaria* [159] and in the firebug *Pyrrhocoris apterus* [160]. Indeed, AKH has the capacity to trigger antioxidant processes [161,162]. In the latter species, mechanical stressors had a similar effect [160], thus strongly arguing for AKH-mediated actions as pivotal element of a widespread stress response. However, honey bees CC contain a lower amount of AKH than other insects (half that of *Gryllus bimaculatus* and *Acheta domesticus*), and AKH has a minor hypertrehalosemic effect in honey bees [163], Thus, the role of AKH in stress responses in honey bees may be less prominent than in other insects. This can possibly be explained by the specific mechanisms honey bees use to mobilize energy (Section 3.3).

### 3.3. Mechanisms of Energy Mobilization in Honey Bees

Increased energy mobilization triggered by hormonal signals plays a very important role in stress responses. In insects, carbohydrates (especially trehalose) are the most important energy source [164]. However, available trehalose is rapidly depleted, and in several insects, a sustained effort such as a long flight requires the release of trehalose from the main energy store—the fat body [109,165]. Consequently, mobilization of energy from energy stores by hormonal factors is an essential part of the stress response. By contrast, honey bees show specific mechanisms for energy mobilization which appear related to their social organization. Strikingly, forager honey bees have fat bodies almost entirely depleted and seem to use the sugar energy reserve carried in their crops to sustain the energetic demands of flight [60,61]. This observation would also explain why foragers express almost no AKH in their CC, and have lower abdominal glycogen stores [166,167].

Honey bees appear to have a quite specific mechanism for regulating hemolymph sugar levels (Figure 3), according to a model proposed by Blatt and Roces [61]. In an energy-demanding situation, trehalose synthesis by the fat body is not fast enough to match rates of trehalose consumption, so circulating trehalose levels decline. This stimulates the passage of nectar from the impermeable crop storage organ to the midgut by the contraction of the proventriculus (gut muscle between the crop and midgut) [62]. From the midgut, sugars are digested, absorbed and more glucose and fructose are transported into the hemolymph. Therefore, upon high metabolic demand, while the trehalose level decreases in the hemolymph, those of fructose and glucose increase, maintaining a stable sugar concentration (Figure 3). Moreover, the honey bee genome sequence suggests the loss of two important insect enzymes converting gluconeogenic substrates to trehalose and glycogen [168], which are both stored in fat body and considered as the primary energy storage molecules in insects. This implies that in the honey bee, regulation of sugar transport from the gut probably plays a more important role in energy balance than the regulation of trehalose release from the fat body. Interestingly, in honey bees injection of CC extracts into the hemolymph has a hypertrehalosemic effect [167], thus candidates for this function are expected to be found in this gland. As discussed above, hormonal candidates performing this role in honey bees might include corazonin, DH-I and possibly AKH (Figure 3).

Hormones activating the mobilization of glucose from the crop by stimulating the proventriculus and midgut remain to be identified. In *Drosophila* [56], it has been suggested that the (neuro)hormones: AST-A and tachykinin, released from secretory cells of the midgut or the nerve cord, might play this role [56] (Figure 3). Tachykinin and tachykinin-related peptides (TRPs) are known in insects to be myostimulatory of the insect midgut muscle [169], and therefore would be good candidates for modulating release of nectar from the honey bee crop. One “tachykinin-like receptor” has been identified in honey bees from sequence homology with *Drosophila* [170], but its function remains unclear, especially as many TRPs have been described and seem to have diverse functions in insects [169]. Nine different TRPs have been localized all along the nervous system of the honey bee [88], but none of them was detected in CC neurosecretory cells. If TRPs act to upregulate metabolism in stress, they would need to be released from the efferent peripheral nerves directly to the proventriculus.

It should be noted that insulin and vitellogenin pathways have also been linked to energy store mobilization and oxidative stress in insects [47,171]. Insulin/insulin-like growth factor signaling (IIS) pathways appears to regulate fat stores in *Drosophila* [171] and confer oxidative stress resistance in *Drosophila* and honey bees [48]. Vitellogenin expression seems to be triggered by IIS pathways [48]. In reproductive females, vitellogenin is a glycolipoprotein stored in the fat body and usually released into the hemolymph before being stored within oocytes. In the sterile honey bee worker, vitellogenin expression is inhibited by JH [172], thus giving foragers a lower amount of vitellogenin (perhaps reinforced by their decrease of fat body mass). However, vitellogenin seems to be protective against oxidative stress, perhaps via certain antioxidant properties, and may account for longevity in reproductive insects, e.g., queen bees [47,48]. Presently, the metabolic role of these molecules needs to be clarified in honey bees in order to be integrated within our model of endocrine regulation of energy sources. 

## 4. Gaps in Knowledge and Urgent Questions

The sections above help build a model of a general stress response in the adult honey bee, as presented in Figure 2. However, as mentioned, in some instances specific data from this particular species are lacking.

### 4.1. What Is the Role of JH in the Stress Response?

JH acts on development and sexual maturation in insects; it is produced in the CC and released under the neural control of brain peptidergic innervation. JH is typically described as the master larval developmental hormone, boosting growth of insects, inhibiting metamorphosis and initiating reproductive traits in adults [173]. Still, JH has been described more recently as a stress hormone in *Drosophila*, as JH levels drop after exposure to various stressors [174]. As JH tends to have long-lasting effects, this hormone may be more likely to be involved in chronic than acute stress response. Whether JH acts as a stress hormone in honey bees is less clear, particularly since, in the adult worker bee, it has a species-specific function: that of a regulator of division of labor. JH titers are low in young nurse bees but higher in foragers. Indeed, pharmacological elevation of JH levels or injection of JH analogs accelerates the onset of foraging of young bees [175,176]. Perhaps because of this, studies of the possible role of JH in stress have thus far given confusing results. Lin *et al.* [34] could not find a consistent change in JH levels after application of various stressors in honey bees, and found a response only if JH levels were initially low (in which case JH levels increased after caging or cold anesthesia). If JH levels were already high, stress seemed to decrease JH levels. These differences probably result from the dynamics of mechanisms for metabolism and recycling of JH when JH levels are very low or very high. As a consequence of this additional complexity, the precise roles of JH in the honey bee stress response are presently unclear, but given the importance of this hormone system, this is certainly an area demanding further study [34].

### 4.2. Can Dopamine Be Considered As a Stress Hormone?

A potential role of hemal dopamine in stress is suggested by work on *Drosophila* as dopamine increased heart rate, while mutations impairing dopamine synthesis had the opposite effect [102]. In cockroaches, one study also found dopamine in the CC [177], thus suggesting a neurohormonal role. In the hemolymph dopamine levels have been rarely quantified in honey bees, but Bateson *et al.* [178], found a decrease in dopamine levels in the head hemolymph of honey bees 30 minutes after a strong vibration stressor. Clearly more work is needed here to explore the possible role of dopamine in endocrine acute stress response.

### 4.3. Neuropeptides in the CC?

Neuropeptides are an emerging area of research, and many members of this large family have never been studied. The neurosecretory cells of the CC contain numerous unstudied peptides [89], which would be good candidates as stress neurohormones. Corazonin, DH, AKH, tachykinins and AST-A have been mentioned previously, but many other peptides might play metabolic roles in the honey bee [167]. In addition, looking at the location of neurohormone receptors is important to understand how the stress system operates. More details on the distribution of neuropeptides and their receptors might highlight their targets, the responses they elicit, as well as the feedback loops regulating the system.

### 4.4. Stress Responses and Immune System

As in vertebrates, the immune response can be affected negatively by stressors. This may be a direct effect of stress hormones (biogenic amines and AKH), as shown in some insect species [179,180]. Depending on the context and the stressor characteristics, the immune response has been shown to be boosted by stressful events or stress hormones [181,182]. This can be understood as a way of maintaining immune equilibrium in a harmful environment [179,183]. In honey bees, stress and immune responses do not seem to have been considered together yet, but recently several detrimental synergistic effects of various combinations of stressors suggest a link between them [184,185,186,187]. As proposed by some of the authors of those studies, such a link may be highly relevant to understand the recent decrease of honey bee populations [186,187].

### 4.5. Task Specialization and Sensitivity to Stress

The high level of sociality and the complex system of division of labor are essential characteristics of honey bees [15]. Here, we propose that at least some elements of the stress response may have been adapted in specific ways to contribute to the evolution of division of labor. During its lifetime, an individual honey bee progresses through a succession of specialized behavioral states whose sequence follows an internal developmental program modulated by various social signals (pheromones) emitted from the colony [188,189]. Honey bees typically begin their adult life undertaking in-hive activities such as brood nursing, cleaning and food storing, then guarding the hive entrance against intruders and predators and finally foraging for food sources (mostly pollen and nectar). The transition to foraging is a major event is a honey bee’s life, and corresponds to multiple changes in hormonal activity, brain circuits, and physiology [188,190].

There has been a lot of research on the mechanisms underlying and organizing this division of labor, which have been shown to involve octopamine, JH and vitellogenin [175,188,191,192]. We suggest here that modulation of stress reactivity may be linked to the evolution of task specialization in honey bees. The fact that octopamine is two to four times more abundant in brains of foragers than those of nurse bees [36] may predispose foragers to attain more easily or rapidly a state of higher energy mobilization. Indeed foragers appear to be the colony members most exposed to stressors: foraging is energetically demanding, and exposes honey bees to more adverse environments (e.g., predators, insecticides) than working within the hive [193]. Elevated brain octopamine levels, as a potential result of chronic stressor exposure [114], may prepare honey bees to cope with the higher stress levels caused by foraging, and the hormonal state of a forager bee may resemble that of nurses bees under chronic stress. 

Further, chronic stressors applied at the colony level and experimental elevation of brain octopamine levels both accelerate the onset of foraging in the honey bee [14,194,195]. A high brain level of octopamine may also make foragers more sensitive to hunger, which could motivate them to gather food.

The forager’s state and number appear to be as a response to colony stress, and foragers are also the behavioral caste exposed to the greatest stress. Therefore, knowing the molecular pathways and physiological mechanisms that regulate chronic and acute stress responses at the individual level are of great interest for developing strategies to improve the health and longevity of honey bee colonies.

## 5. Conclusions

The model developed here describes a general stress response in honey bees. It provides a framework to facilitate our understanding of how honey bees can respond to stressors, and is also aimed at stimulating research to improve our knowledge of the physiological pathways involved.

Our comparison of vertebrate and honey bee stress response pathways suggests a parallel organizational structure in the two groups, including regulation of arousal and cognitive functions in the brain by catecholamines, coupled with neurohormonal signals stimulating energy mobilization in the periphery. Yet, the extent to which the stress response pathways are evolutionarily conserved remains unclear. Some elements, like CRH-BP, may offer examples of conservation of function, but others, particularly neuropeptide hormones, are likely to be specific to insects or invertebrates. In this regard, it is worth noting that several key neuropeptides cited here (AKH, tachykinin, DH) are among the most highly conserved neuropeptides among insect species, perhaps indicating the operation of strong stabilizing selection [196].

In this review, we also highlighted the aspects of the stress response that appear to be specific to honey bees as a result of their peculiar social organization. Specifically, we have summarized particular mechanisms enabling an increase of glucose from the crop. 

Finally, pursuing studies on stress in honey bees is essential for developing standard methods to assess stress in this insect of major economical importance. Relevant and robust criteria to evaluate stress symptoms would be useful as basic indicators of health in honey bees, and the development of standardized assays would improve risk assessment for pesticide and other agricultural practices on honey bee populations. Thus, knowing more about stress in honey bees is now crucial to design strategies for the protection of this fragile, but ecologically and economically important, insect. 

## References

[1] Selye H. (1956). The Stress of Life.

[2] Chrousos G.P. (2009). Stress and disorders of the stress system. Nat. Rev. Endocrinol..

[3] Chrousos G.P. (1998). Stressors, Stress, and Neuroendocrine Integration of the Adaptive Response: The 1997 Hans Selye Memorial Lecture. Ann. NY Acad. Sci..

[4] Selye H. (1936). A syndrome produced by diverse nocuous agents. Nature.

[5] McEwen B.S. (2009). The brain is the central organ of stress and adaptation. NeuroImage.

[6] Boerjan B., Verleyen P., Huybrechts J., Schoofs L., de Loof A. (2010). In search for a common denominator for the diverse functions of arthropod corazonin: A role in the physiology of stress?. Gen. Comp. Endocr..

[7] Ivanovic J., Ivanovic J., Jankovic-Hlandni M. (1991). Metabolic response to stressor. Hormones and Metabolism in Insect stress.

[8] Roeder T. (2005). Tyramine and octopamine: Ruling Behavior and Metabolism. Annu. Rev. Entomol..

[9] Johnson E.C., White M.P., Pfaff D.W., Arnold A.P., Fahrbach S.E., Etgen A.M., Rubin R.T. (2009). Stressed-Out Insects: Hormonal Actions and Behavioral Modifications. Horm. Brain Behav..

[10] VanEngelsdorp D., Evans J.D., Saegerman C., Mullin C., Haubruge E., Nguyen B.K., Frazier M., Frazier J., Cox-Foster D., Chen Y. (2009). Colony Collapse Disorder: A Descriptive Study. PLoS One.

[11] Ratnieks F.L.W., Carreck N.L. (2010). Clarity on Honey Bee Collapse?. Science.

[12] Neumann P., Carreck N. (2010). Honey bee colony losses. J. Apic. Res..

[13] Oldroyd B.P. (2007). What’s Killing American Honey Bees?. PLoS Biol..

[14] Khoury D.S., Myerscough M.R., Barron A.B. (2011). A Quantitative Model of Honey Bee Colony Population Dynamics. PLoS One.

[15] Wilson E.O. (1971). The insect societies (Belknap Press).

[16] Bamberger C.M., Schulte H.M., Chrousos G.P. (1996). Molecular Determinants of Glucocorticoid Receptor Function and Tissue Sensitivity to Glucocorticoids. Endocr. Rev..

[17] McEwen B.S. (2000). The neurobiology of stress: From serendipity to clinical relevance. Brain Res..

[18] Stratakis C.A., Chrousos G.P. (1995). Neuroendocrinology and Pathophysiology of the Stress System. Ann. NY Acad. Sci..

[19] Santoro M.G. (2000). Heat shock factors and the control of the stress response. Biochem. Pharmacol..

[20] Takeda K., Noguchi T., Naguro I., Ichijo H. (2008). Apoptosis Signal-Regulating Kinase 1 in Stress and Immune Response. Annu. Rev. Pharmacol. Toxicol..

[21] Feder M.E., Hofmann G.E. (1999). Heat-Shock Proteins, molecular chaperones, and the stress response: evolutionary and ecological physiology. Annu. Rev. Physiol..

[22] Gibney E., Gault J., Williams J. (2001). The use of stress proteins as a biomarker of sub-lethal toxicity: Induction of heat shock protein 70 by 2-isobutyl piperidine and transition metals at sub-lethal concentrations. Biomarkers.

[23] Nazir A., Saxena D.K., Kar Chowdhuri D. (2003). Induction of hsp70 in transgenic Drosophila: Biomarker of exposure against phthalimide group of chemicals. Biochim. Biophys. Acta Gen. Subj..

[24] Stetler R.A., Gan Y., Zhang W., Liou A.K., Gao Y., Cao G., Chen J. (2010). Heat shock proteins: Cellular and molecular mechanisms in the central nervous system. Prog. Neurobiol..

[25] Ulrich-Lai Y.M., Herman J.P. (2009). Neural regulation of endocrine and autonomic stress responses. Nat. Rev. Neurosci..

[26] Cannon W.B. (1915). Bodily Changes in Pain, Hunger, Fear and Range.

[27] Gregorc A., Bowen I.D. (1999). *In situ* localization of heat-shock and histone proteins in honey-bee (*Apis mellifera* l.) larvae infected with paenibacillus larvae. Cell Biol. Int..

[28] Elekonich M. (2009). Extreme thermotolerance and behavioral induction of 70-kDa heat shock proteins and their encoding genes in honey bees. Cell Stress Chaperones.

[29] Severson D.W., Erickson E.H., Williamson J.L., Aiken J.M. (1990). Heat stress induced enhancement of heat shock protein gene activity in the honey bee (Apis mellifera). Cell Mol. Life Sci..

[30] Li Y., Zhang L., Kang M., Guo X., Baohua X. (2012). AccERK2, a map kinase gene from *Apis cerana cerana*, plays roles in stress responses, developmental processes, and the nervous system. Arch. Insect Biochem. Physiol..

[31] Hranitz J.M., Abramson C.I., Carter R.P. (2010). Ethanol increases HSP70 concentrations in honeybee (*Apis mellifer*a L.) brain tissue. Alcohol.

[32] Corona M., Hughes K.A., Weaver D.B., Robinson G.E. (2005). Gene expression patterns associated with queen honey bee longevity. Mech. Ageing Dev..

[33] Duell M.E., Abramson C.I., Wells H., Aptes T.E., Hall N.M., Pendergraft L.J., Zuniga E.M., Oruç H.H., Sorucu A., Çakmak I. (2012). An Integrative Model of Cellular Stress and Environmental Stressors in the Honey Bee. Insects.

[34] Lin H., Dusset C., Huang Z.Y. (2004). Short-term changes in juvenile hormone titers in honey bee workers due to stress. Apidologie.

[35] Chen Y.L., Hung Y.S., Yang E.C. (2008). Biogenic amine levels change in the brains of stressed honeybees. Arch. Insect Biochem. Physiol..

[36] Harris J.W., Woodring J. (1992). Effects of stress, age, season, and source colony on levels of octopamine, dopamine and serotonin in the honey bee (*Apis mellifera* L.) brain. J. Insect Physiol..

[37] Liu L., Yu X., Meng F., Guo X., Xu B. (2011). Identification and characterization of a novel corticotropin-releasing hormone-binding protein (CRH-BP) gene from Chinese honeybee (*Apis cerana cerana*). Arch. Insect Biochem. Physiol..

[38] Lenoir J.C., Laloi D., Dechaume-Moncharmont F.X., Solignac M., Pham M.H. (2006). Intra-colonial variation of the sting extension response in the honey bee *Apis mellifera*. Insectes Soc..

[39] Uribe-Rubio J., Guzmán-Novoa E., Vázquez-Peláez C., Hunt G. (2008). Genotype, Task Specialization, and Nest Environment Influence the Stinging Response Thresholds of Individual Africanized and European Honeybees to Electrical Stimulation. Behav. Genet..

[40] Balderrama N., Diaz H., Sequeda A., Nunez J., Maldonato H., Menzel R., Mercer A. (1987). Behavioral and Pharmacological Analysis of the Stinging Response in Africanized and Italian Bees. Neurobiology and behavior of honeybees.

[41] Balderrama N., Núñez J., Guerrieri F., Giurfa M. (2002). Different functions of two alarm substances in the honeybee. J. Comp. Physiol. A.

[42] Roussel E., Carcaud J., Sandoz J.C., Giurfa M. (2009). Reappraising Social Insect Behavior through Aversive Responsiveness and Learning. PLoS One.

[43] Núñez J., Almeida L., Balderrama N., Giurfa M. (1997). Alarm Pheromone Induces Stress Analgesia via an Opioid System in the Honeybee. Physiol. Behav..

[44] Núñez J., Maldonado H., Miralto A., Balderrama N. (1983). The stinging response of the honeybee: Effects of morphine, naloxone and some opioid peptides. Pharmacol. Biochem. Behav..

[45] Hladun K.R., Smith B.H., Mustard J.A., Morton R.R., Trumble J.T. (2012). Selenium Toxicity to Honey Bee (*Apis mellifera* L.) Pollinators: Effects on Behaviors and Survival. PLoS ONE.

[46] Amdam G.V., Fennern E., Baker N., Rascon B. (2010). Honeybee Associative Learning Performance and Metabolic Stress Resilience Are Positively Associated. PLoS One.

[47] Seehuus S.C., Norberg K., Gimsa U., Krekling T., Amdam G.V. (2006). Reproductive protein protects functionally sterile honey bee workers from oxidative stress. Proc. Natl. Acad. Sci. USA.

[48] Corona M., Velarde R.A., Remolina S., Moran-Lauter A., Wang Y., Hughes K.A., Robinson G.E. (2007). Vitellogenin, juvenile hormone, insulin signaling, and queen honey bee longevity. Proc. Natl. Acad. Sci. USA.

[49] Corbet S.A. (1991). A Fresh Look at the Arousal Syndrome of Insects. Adv. Insect Physiol..

[50] Farooqui T. (2012). Review of octopamine in insect nervous systems. Open Access Insect Physiol..

[51] Papaefthimiou C., Theophilidis G. (2011). Octopamine, a single modulator with double action on the heart of two insect species (*Apis mellifera macedonica* and *Bactrocera oleae*): Acceleration *vs.* inhibition. J. Insect Physiol..

[52] Pflüger H.J., Duch C., Heidel E. (2004). Neuromodulatory octopaminergic neurones and their functions during insect motor behaviour. Acta Biol. Hung..

[53] Mustard J.A., Pham P.M., Smith B.H. (2010). Modulation of motor behavior by dopamine and the D1-like dopamine receptor AmDOP2 in the honey bee. J. Insect Physiol..

[54] Kodrík D. (2008). Adipokinetic hormone functions that are not associated with insect flight. Physiol. Entomol..

[55] Coast G.M., Orchard I., Phillips J.E., Schooley D.A. (2002). Insect diuretic and antidiuretic hormones. Adv. Insect Physiol..

[56] Veenstra J.A. (2009). Does corazonin signal nutritional stress in insects?. Insect Biochem. Mol. Biol..

[57] Davenport A.P., Evans P.D. (1984). Stress-induced changes in the octopamine levels of insect haemolymph. Insect Biochem..

[58] Kreissl S., Eichmüller S., Bicker G., Rapus J., Eckert M. (1994). Octopamine-like immunoreactivity in the brain and subesophageal ganglion of the honeybee. J. Comp. Neurol..

[59] Verlinden H., Vleugels R., Marchal E., Badisco L., Pflüger H.J., Blenau W., Broeck J.V. (2010). The role of octopamine in locusts and other arthropods. J. Insect Physiol..

[60] Crailsheim K. (1988). Intestinal transport of sugars in the honeybee (*Apis mellifera* L.). J. Insect Physiol..

[61] Blatt J., Roces F. (2001). Haemolymph sugar levels in foraging honeybees (*Apis mellifera carnica*): Dependence on metabolic rate and *in vivo* measurement of maximal rates of trehalose synthesis. J. Exp. Biol..

[62] Blatt J., Roces F. (2002). The control of the proventriculus in the honeybee (*Apis mellifera carnica* L.) II. Feedback mechanisms. J. Insect Physiol..

[63] Kvetnansky R., Sabban E.L., Palkovits M. (2009). Catecholaminergic Systems in Stress: Structural and Molecular Genetic Approaches. Physiol. Rev..

[64] Roeder T. (1999). Octopamine in invertebrates. Prog. Neurobiol..

[65] Bicker G., Menzel R. (1989). Chemical codes for the control of behaviour in arthropods. Nature.

[66] Evans P., Maqueira B. (2005). Insect octopamine receptors: A new classification scheme based on studies of cloned Drosophila G-protein coupled receptors. Invert Neurosci..

[67] Scheiner R., Baumann A., Blenau W. (2006). Aminergic control and modulation of honeybee behaviour. Curr. Neuropharmacol..

[68] Sombati S., Hoyle G. (1984). Generation of specific behaviors in a locust by local release into neuropil of the natural neuromodulator octopamine. J. Neurobiol..

[69] Andretic R., van Swinderen B., Greenspan R.J. (2005). Dopaminergic Modulation of Arousal in Drosophila. Curr. Biol..

[70] Van Swinderen B., Andretic R. (2011). Dopamine in Drosophila: Setting arousal thresholds in a miniature brain. Proc. R. Soc. B.

[71] Crocker A., Shahidullah M., Levitan I.B., Sehgal A. (2010). Identification of a Neural Circuit that Underlies the Effects of Octopamine on Sleep:Wake Behavior. Neuron.

[72] Lebestky T., Chang J.S.C., Dankert H., Zelnik L., Kim Y.C., Han K.A., Wolf F.W., Perona P., Anderson D.J. (2009). Two Different Forms of Arousal in Drosophila Are Oppositely Regulated by the Dopamine D1 Receptor Ortholog DopR via Distinct Neural Circuits. Neuron.

[73] Fussnecker B.L., Smith B.H., Mustard J.A. (2006). Octopamine and tyramine influence the behavioral profile of locomotor activity in the honey bee (*Apis mellifera*). J. Insect Physiol..

[74] Pribbenow B., Erber J. (1996). Modulation of Antennal Scanning in the Honeybee by Sucrose Stimuli, Serotonin, and Octopamine: Behavior and Electrophysiology. Neurobiol. Learn. Mem..

[75] Barron A.B., Maleszka R., Vander Meer R.K., Robinson G.E. (2007). Octopamine modulates honey bee dance behavior. Proc. Natl. Acad. Sci. USA.

[76] McQuillan H., Barron A., Mercer A. (2012). Age- and behaviour-related changes in the expression of biogenic amine receptor genes in the antennae of honey bees (*Apis mellifera*). J. Comp. Physiol. A.

[77] Menzel R., Heyne A., Kinzel C., Gerber B., Fiala A. (1999). Pharmacological dissociation between the reinforcing, sensitizing, and response-releasing functions of reward in honeybee classical conditioning. Behav. Neurosci..

[78] Heisenberg M. (2003). Mushroom body memoir: From maps to models. Nat. Rev. Neurosci..

[79] Vergoz V., Roussel E., Sandoz J.C., Giurfa M. (2007). Aversive learning in honeybees revealed by the olfactory conditioning of the sting extension reflex. PLoS One.

[80] Agarwal M., Giannoni Guzmán M., Morales-Matos C., Del Valle Díaz R.A., Abramson C.I., Giray T. (2011). Dopamine and Octopamine Influence Avoidance Learning of Honey Bees in a Place Preference Assay. PLoS One.

[81] Roozendaal B.F., McGaugh J.L. (2011). Memory modulation. Behav. Neurosci..

[82] Huising M.O., Metz J.R., van Schooten C., Taverne-Thiele A.J., Hermsen T.,  Kemenade B.M., Flik G. (2004). Structural characterisation of a cyprinid (C*yprinus carpio* L.) CRH, CRH-BP and CRH-R1, and the role of these proteins in the acute stress response. J. Mol. Endocrinol..

[83] Westphal N.J., Seasholtz A.F. (2006). CRH-BP: The regulation and function of a phylogenetically conserved binding protein. Front. Biosci..

[84] Chang C.L., Hsu S.Y.T. (2004). Ancient evolution of stress-regulating peptides in vertebrates. Peptides.

[85] Huising M.O., Flik G. (2005). The Remarkable Conservation of Corticotropin-Releasing Hormone (CRH)-Binding Protein in the Honeybee (*Apis mellifera*) Dates the CRH System to a Common Ancestor of Insects and Vertebrates. Endocrinology.

[86] Lovejoy D.A., Balment R.J. (1999). Evolution and Physiology of the Corticotropin-Releasing Factor (CRF) Family of Neuropeptides in Vertebrates. Gen. Comp. Endocr..

[87] Zandawala M. (2012). Calcitonin-like diuretic hormones in insects. Insect Biochem. Mol. Biol..

[88] Boerjan B., Cardoen D., Bogaerts A., Landuyt B., Schoofs L., Verleyen P. (2010). Mass spectrometric profiling of (neuro)-peptides in the worker honeybee, *Apis mellifera*. Neuropharmacology.

[89] Scharrer B. (1967). The neurosecretory neuron in neuroendocrine regulatory mechanisms. Am. Zool..

[90] De Loof A., Lindemans M., Liu F., de Groef B., Schoofs L. (2012). Endocrine archeology: Do insects retain ancestrally inherited counterparts of the vertebrate releasing hormones GnRH, GHRH, TRH, and CRF?. G. Comp. Endocr..

[91] Malamud J.G., Mizisin A.P., Josephson R.K. (1988). The effects of octopamine on contraction kinetics and power output of a locust flight muscle. J. Comp. Physiol. A.

[92] Orchard I., Lange A.B. (1985). Evidence for octopaminergic modulation of an insect visceral muscle. J. Neurobiol..

[93] Luffy D., Dorn A. (1992). Immunohistochemical demonstration in the stomatogastric nervous system and effects of putative neurotransmitters on the motility of the isolated midgut of the stick insect, *Carausius morosus*. J. Insect Physiol..

[94] Lange A.B., Orchard I. (1986). Identified octopaminergic neurons modulate contractions of locust visceral muscle via adenosine 3',5'-monophosphate (cyclic AMP). Brain Res..

[95] Orchard I., Lange A.B. (1987). Cockroach oviducts: The presence and release of octopamine and proctolin. J. Insect Physiol..

[96] Monastirioti M. (2003). Distinct octopamine cell population residing in the CNS abdominal ganglion controls ovulation in Drosophila melanogaster. Dev. Biol..

[97] Avila F.W., Bloch Qazi M.C., Rubinstein C.D., Wolfner M.F. (2012). A requirement for the neuromodulators octopamine and tyramine in Drosophila melanogaster female sperm storage. Proc. Natl. Acad. Sci. USA.

[98] Stevenson P.A., Pflüger H.J., Eckert M., Rapus J. (1994). Octopamine-like immunoreactive neurones in locust genital abdominal ganglia. Cell Tissue Res..

[99] Prier K.R., Beckman O.H., Tublitz N.J. (1994). Modulating a modulator: Biogenic amines at subthreshold levels potentiate peptide-mediated cardioexcitation of the heart of the tobacco hawkmoth *Manduca sexta*. J. Exp. Biol..

[100] Hertel W., Penzlin H. (1992). Function and modulation of the antennal heart of *Periplaneta americana* (L.). Acta Biol. Hung..

[101] Collins C., Miller T. (1977). Studies on the action of biogenic amines on cockroach heart. J. Exp. Biol..

[102] Johnson E., Ringo J., Dowse H. (1997). Modulation of Drosophila heartbeat by neurotransmitters. J. Comp. Physiol. B.

[103] Zeng H., Loughton B.G., Jennings K.R. (1996). Tissue specific transduction systems for octopamine in the locust (*Locusta migratoria*). J. Insect Physiol..

[104] Farooqui T. (2007). Octopamine-Mediated Neuromodulation of Insect Senses. Neurochem. Res..

[105] Downer R.G.H. (1979). Trehalose production in isolated fat body of the american cockroach, Periplaneta americana. Comp. Biochem. Physiol. C Comp. Pharmacol..

[106] Gole J.W.D., Downer R.G.H. (1979). Elevation of adenosine 3',5'-monophosphate by octopamine in fat body of the american cockroach, *Periplaneta americana* L. Comp. Biochem. Physiol. C Comp. Pharmacol..

[107] Orchard I., Carlisle J.A., Loughton B.G., Gole J.W.D., Downer R.G.H. (1982). *In vitro* studies on the effects of octopamine on locust fat body. Gen. Comp. Endocr..

[108] Meyer-Fernandes J.R., Gondim K.C., Wells M.A. (2000). Developmental changes in the response of larval *Manduca sexta* fat body glycogen phosphorylase to starvation, stress and octopamine. Insect Biochem. Mol. Biol..

[109] Arrese E.L., Soulages J.L. (2010). Insect Fat Body: Energy, Metabolism, and Regulation. Annu. Rev. Entomol..

[110] Goosey M.W., Candy D.J. (1982). The release and removal of octopamine by tissues of the locust Schistocerca americana gregaria. Insect Biochem..

[111] Martin R.J., Jahagirdar A.P., Downer R.G.H. (1989). Partial characterization of *N*-acetyltransferase activity from cerebral ganglia and malpighian tubules of *Periplaneta americana*. Insect Biochem..

[112] David J.C., Lafon-Cazal M. (1979). Octopamine distribution in the Locusta migratoria nervous and non-nervous, systems. Comp. Biochem. Physiol. C Comp. Pharmacol..

[113] Lam F., McNeil J.N., Donly C. (2012). Octopamine receptor gene expression in three lepidopteran species of insect. Peptides..

[114] Adamo S.A., Baker J.L. (2011). Conserved features of chronic stress across phyla: The effects of long-term stress on behavior and the concentration of the neurohormone octopamine in the cricket, *Gryllus texensis*. Horm. Behav..

[115] Duch C., Pflüger H.J. (1999). DUM neurons in locust flight: A model system for amine-mediated peripheral adjustments to the requirements of a central motor program. J. Comp. Physiol. A.

[116] Orchard I., Ramirez J.M., Lange A.B. (1993). A multifunctional role for octopamine in locust flight. Annu. Rev. Entomol..

[117] Bellah K.L., Fitch G.K., Kammer A.E. (1984). A central action of octopamine on ventilation frequency in *Corydalus cornutus*. J. Exp. Zool..

[118] Ramirez J.M., Pearson K.G. (1991). Octopamine induces bursting and plateau potentials in insect neurones. Brain Res..

[119] Huang J., Wu S.F., Li X.H., Adamo S.A., Ye G.Y. (2012). The characterization of a concentration-sensitive adrenergic-like octopamine receptor found on insect immune cells and its possible role in mediating stress hormone effects on immune function. Brain Behav. Immun..

[120] Bicker G. (1999). Biogenic amines in the brain of the honeybee: Cellular distribution, development, and behavioral functions. Micros. Res. Tech..

[121] Sinakevitch I., Niwa M., Strausfeld N.J. (2005). Octopamine-like immunoreactivity in the honey bee and cockroach: Comparable organization in the brain and subesophageal ganglion. J. Comp. Neurol..

[122] Schröter U., Malun D., Menzel R. (2007). Innervation pattern of suboesophageal ventral unpaired median neurones in the honeybee brain. Cell Tissue Res..

[123] Stevenson P.A., Sporhase-Eichmann U. (1995). Localization of octopaminergic neurones in insects. Comp. Biochem. Physiol. Part A Mol. Integr. Physiol..

[124] Bräunig P. (1997). The peripheral branching pattern of identified dorsal unpaired median (DUM) neurones of the locust. Cell Tissue Res..

[125] Bräunig P., Pflüger H.J. (2001). The unpaired median neurons of insects. Adv. Insect Physiol..

[126] Field L.H., Duch C., Pflüger H.J. (2008). Responses of efferent octopaminergic thoracic unpaired median neurons in the locust to visual and mechanosensory signals. J. Insect Physiol..

[127] Bräunig P. (1995). Dorsal unpaired median (DUM) neurones with neurohaemal functions in the locust, *Locusta migratoria*. Acta Biol. Hung..

[128] Bräunig P., Stevenson P.A., Evans P.D. (1994). A locust octopamine-immunoreactive dorsal unpaired median neurone forming terminal networks on sympathetic nerves. J. Exp. Biol..

[129] Veenstra J.A. (1989). Isolation and structure of corazonin, a cardioactive peptide from the American cockroach. FEBS Lett..

[130] Tawfik A.I., Tanaka S., de Loof A., Schoofs L., Baggerman G., Waelkens E., Derua R., Milner Y., Yerushalmi Y., Pener M.P. (1999). Identification of the gregarization-associated dark-pigmentotropin in locusts through an albino mutant. Proc. Natl. Acad. Sci. USA.

[131] Kim Y.J., Spalovska-Valachova I., Cho K.H., Zitnanova I., Park Y., Adams M.E., Zitnan D. (2004). Corazonin receptor signaling in ecdysis initiation. Proc. Natl. Acad. Sci. USA.

[132] Roller L., Tanaka S., Kimura K., Satake H., Tanaka Y. (2006). Molecular cloning of [Thr4], [His7]-corazonin (Apime-corazonin) and its distribution in the central nervous system of the honey bee *Apis mellifera* (Hymenoptera: Apidae). Appl. Entomol. Zool..

[133] Verleyen P., Baggerman G., Mertens I., Vandersmissen T., Huybrechts J., Lommel A.V., de Loof A., Schoofs L. (2006). Cloning and characterization of a third isoform of corazonin in the honey bee *Apis mellifera*. Peptides.

[134] Chintapalli V.R., Wang J., Dow J.A.T. (2007). Using FlyAtlas to identify better Drosophila melanogaster models of human disease. Nat. Genet..

[135] Johnson E.C., Shafer O.T., Trigg J.S., Park J., Schooley D.A., Dow J.A., Taghert P.H. (2005). A novel diuretic hormone receptor in Drosophila: Evidence for conservation of CGRP signaling. J. Exp. Biol..

[136] Park Y., Kim Y.J., Adams M.E. (2002). Identification of G protein-coupled receptors for Drosophila PRXamide peptides, CCAP, Corazonin, and AKH supports a theory of lignad-receptor coevolution. Proc. Natl. Acad. Sci. USA.

[137] Hansen K.K., Stafflinger E., Schneider M., Hauser F., Cazzamali G., Williamson M., Kollmann M., Schachtner J., Grimmelikhuijzen C.J.P. (2010). Discovery of a Novel Insect Neuropeptide Signaling System Closely Related to the Insect Adipokinetic Hormone and Corazonin Hormonal Systems. J. Biol. Chem..

[138] Bendena W.G., Donly B.C., Tobe S.S. (1999). Allatostatins: A Growing Family of Neuropeptides with Structural and Functional Diversity. Ann. NY Acad. Sci..

[139] Stay B., Tobe S.S. (2007). The role of allatostatins in juvenile hormone synthesis in insects and crustaceans. Annu. Rev. Entomol..

[140] Audsley N., Weaver R.J. (2009). Neuropeptides associated with the regulation of feeding in insects. Gen. Comp. Endocr..

[141] Veenstra J.A. (2009). Allatostatin C and its paralog allatostatin double C: The arthropod somatostatins. Insect Biochem. Mol. Biol..

[142] Meyering-Vos M., Woodring J. (2008). A-type allatostatins and sulfakinins as satiety effectors in the Mediterranean field cricket *Gryllus bimaculatus*. Mitt. Dtsch. Ges. Allg. Angew. Entomol..

[143] Veenstra J., Agricola H.J., Sellami A. (2008). Regulatory peptides in fruit fly midgut. Cell Tissue Res..

[144] Wilson C.H., Christie A.E. (2010). Distribution of C-type allatostatin (C-AST)-like immunoreactivity in the central nervous system of the copepod *Calanus finmarchicus*. Gen. Comp. Endocr..

[145] Robertson L., Rodriguez E.P., Lange A.B. (2012). The neural and peptidergic control of gut contraction in Locusta migratoria: The effect of an FGLa/AST. J. Exp. Biol..

[146] Wang C., Chin-Sang I., Bendena W.G. (2012). The FGLamide-Allatostatins Influence Foraging Behavior in *Drosophila melanogaster*. PLoS One.

[147] Audsley N., Matthews J., Nachman R.J., Weaver R.J. (2008). Transepithelial flux of an allatostatin and analogs across the anterior midgut of *Manduca sexta* larvae *in vitro*. Peptides.

[148] Mayoral J.G., Nouzova M., Brockhoff A., Goodwin M., Hernandez-Martinez S., Richter D., Meyerhof W., Noriega F.G. (2010). Allatostatin-C receptors in mosquitoes. Peptides.

[149] Hergarden A.C., Tayler T.D., Anderson D.J. (2012). Allatostatin-A neurons inhibit feeding behavior in adult *Drosophila*. Proc. Natl. Acad. Sci. USA.

[150] Kreissl S., Strasser C., Galizia C.G. (2010). Allatostatin immunoreactivity in the honeybee brain. J. Comp. Neurol..

[151] Gade G., Auerswald L. (2003). Mode of action of neuropeptides from the adipokinetic hormone family. Gen. Comp. Endocr..

[152] Bharucha K.N., Tarr P., Zipursky S.L. (2008). A glucagon-like endocrine pathway in Drosophila modulates both lipid and carbohydrate homeostasis. J. Exp. Biol..

[153] Isabel G., Martin J.R., Chidami S., Veenstra J.A., Rosay P. (2005). AKH-producing neuroendocrine cell ablation decreases trehalose and induces behavioral changes in *Drosophila*. Am. J. Physiol. Regul. Integr. Comp. Physiol..

[154] Lee G., Park J.H. (2004). Hemolymph sugar homeostasis and starvation-induced hyperactivity affected by genetic manipulations of the adipokinetic hormone-encoding gene in Drosophila melanogaster. Genetics.

[155] Wicher D. (2007). Metabolic Regulation and Behavior: How Hunger Produces Arousal—An Insect Study. Endocr. Metab. Immune Disord. Drug Targets.

[156] Pannabecker T., Orchard I. (1986). Octopamine and cyclic AMP mediate release of adipokinetic hormone I and II from isolated locust neuroendocrine tissue. Mol. Cell Endocrinol..

[157] Passier P.C.C.M., Vullings H.G.B., Diederen J.H.B., van der Horst D.J. (1995). Modulatory Effects of Biogenic Amines on Adipokinetic Hormone Secretion from Locust Corpora Cardiaca *in Vitro*. Gen. Comp. Endocr..

[158] Pannabecker T., Orchard I. (1987). Regulation of adipokinetic hormone release from locust neuroendocrine tissue: Participation of calcium and cyclic AMP. Brain Res..

[159] Candy D.J. (2002). Adipokinetic hormones concentrations in the haemolymph of Schistocerca gregaria, measured by radioimmunoassay. Insect Biochem. Mol. Biol..

[160] Kodrík D., Socha R. (2005). The effect of insecticide on adipokinetic hormone titre in the insect body. Pest Manage. Sci..

[161] Velki M., Kodrík D., Vecera J., Hackenberger B.K., Socha R. (2011). Oxidative stress elicited by insecticides: A role for the adipokinetic hormone. Gen. Comp. Endocr..

[162] Večeřa J., Krishnan N., Mithöfer A., Vogel H., Kodrík D. (2012). Adipokinetic hormone-induced antioxidant response in *Spodoptera littoralis*. Comp. Biochem. Physiol. Part C Toxicol. Pharmacol..

[163] Lorenz M.W., Kellner R., Woodring J., Hoffmann K.H., Gade G. (1999). Hypertrehalosaemic peptides in the honeybee (*Apis mellifera*): Purification, identification and function. J. Insect Physiol..

[164] Thompson S.N. (2003). Trehalose—The Insect “Blood” Sugar. Adv. Insect Physiol..

[165] Van der Horst D.J. (2003). Insect adipokinetic hormones: Release and integration of flight energy metabolism. Comp. Biochem. Physiol. Part B Biochem. Mol. Biol..

[166] Panzenbock U., Crailsheim K. (1997). Glycogen in honeybee queens, workers and drones (*Apis mellifera carnica* Pollm.). J. Insect Physiol..

[167] Woodring J., Das S., Gade G. (1994). Hypertrehalosemic factors from the corpora cardiaca of the honeybee (*Apis mellifera*) and the paper wasp (*Polistes exclamans*). J. Insect Physiol..

[168] Kunieda T., Fujiyuki T., Kucharski R., Foret S., Ament S.A., Toth A.L., Ohashi K., Takeuchi H., Kamikouchi A., Kage E. (2006). Carbohydrate metabolism genes and pathways in insects: insights from the honey bee genome. Insect Mol. Biol..

[169] Nassel D.R. (1999). Tachykinin-related peptides in invertebrates: a review. Peptides.

[170] Hauser F., Cazzamali G., Williamson M., Blenau W., Grimmelikhuijzen C.J.P. (2006). A review of neurohormone GPCRs present in the fruitfly Drosophila melanogaster and the honey bee *Apis mellifera*. Prog. Neurobiol..

[171] Broughton S.J., Piper M.D.W., Ikeya T., Bass T.M., Jacobson J., Driege Y., Martinez P., Hafen E., Withers D.J., Leevers S.J. (2005). Longer lifespan, altered metabolism, and stress resistance in *Drosophila* from ablation of cells making insulin-like ligands. Proc. Natl. Acad. Sci. USA.

[172] Pinto L.Z., Bitondi M.M.G., Simões Z.L.P. (2000). Inhibition of vitellogenin synthesis in *Apis mellifera* workers by a juvenile hormone analogue, pyriproxyfen. J. Insect Physiol..

[173] Riddiford L.M. (2012). How does juvenile hormone control insect metamorphosis and reproduction?. Gen. Comp. Endocr..

[174] Gruntenko N.E., Bogomolova E.V., Adonyeva N.V., Karpova E.K., Menshanov P.N., Alekseev A.A., Romanova I.V., Li S., Rauschenbach I.Y. (2012). Decrease in juvenile hormone level as a result of genetic ablation of the Corpus allatum cells affects the synthesis and metabolism of stress related hormones in *Drosophila*. J. Insect Physiol..

[175] Robinson G.E. (1987). Regulation of honey bee age polyethism by juvenile hormone. Behav. Ecol. Sociobiol..

[176] Schulz D.J., Sullivan J.P., Robinson G.E. (2002). Juvenile Hormone and Octopamine in the Regulation of Division of Labor in Honey Bee Colonies. Horm. Behav..

[177] Shimizu T., Mihara M., Takeda N. (1991). High-performance liquid chromatography of biogenic amines in the corpus cardiacum of the American cockroach, *Periplaneta americana*. J. Chromatogr. A.

[178] Bateson M., Desire S., Gartside S.E., Wright G.A. (2011). Agitated Honeybees Exhibit Pessimistic Cognitive Biases. Curr. Biol..

[179] Adamo S.A. (2012). The effects of the stress response on immune function in invertebrates: An evolutionary perspective on an ancient connection. Horm. Behav..

[180] Adamo S.A., Parsons N.M. (2006). The emergency life-history stage and immunity in the cricket, *Gryllus texensis*. Anim. Behav..

[181] Baines D., DeSantis T., Downer R.G.H. (1992). Octopamine and 5-hydroxytryptamine enhance the phagocytic and nodule formation activities of cockroach (*Periplaneta americana*) haemocytes. J. Insect Physiol..

[182] Mowlds P., Barron A., Kavanagh K. (2008). Physical stress primes the immune response of Galleria mellonella larvae to infection by Candida albicans. Microbes Infect..

[183] Adamo S.A., Roberts J.L., Easy R.H., Ross N.W. (2008). Competition between immune function and lipid transport for the protein apolipophorin III leads to stress-induced immunosuppression in crickets. J. Exp. Biol..

[184] Alaux C., Brunet J.L., Dussaubat C., Mondet F., Tchamitchan S., Cousin M., Brillard J., Baldy A., Belzunces L.P., Le Conte Y. (2010). Interactions between Nosema microspores and a neonicotinoid weaken honeybees (*Apis mellifera*). Environ. Microbiol..

[185] Aufauvre J., Biron D.G., Vidau C., Fontbonne R., Roudel M., Diogon M., Viguès B., Belzunces L.P., Delbac F., Blot N. (2012). Parasite-insecticide interactions: A case study of Nosema ceranae and fipronil synergy on honeybee. Sci. Rep..

[186] Köhler A., Pirk C.W.W., Nicolson S.W. (2012). Simultaneous stressors: Interactive effects of an immune challenge and dietary toxin can be detrimental to honeybees. J. Insect Physiol..

[187] Vidau C., Diogon M., Aufauvre J., Fontbonne R., Viguès B., Brunet J.L., Texier C., Biron D.G., Blot N., El Alaoui H. (2011). Exposure to Sublethal Doses of Fipronil and Thiacloprid Highly Increases Mortality of Honeybees Previously Infected by *Nosema ceranae*. PLoS ONE.

[188] Robinson G.E. (1992). Regulation of Division of Labor in Insect Societies. Annu. Rev. Entomol..

[189] Slessor K., Winston M., Le Conte Y. (2005). Pheromone Communication in the Honeybee (*Apis mellifera* L.). J. Chem. Ecol..

[190] Winston M.L. (1987). The biology of the honey bee.

[191] Wagener-Hulme C., Kuehn J.C., Schulz D.J., Robinson G.E. (1999). Biogenic amines and division of labor in honey bee colonies. J. Comp. Physiol. A.

[192] Fahrbach S.E., Robinson G.E. (1996). Juvenile hormone, behavioral maturation, and brain structure in the honey bee. Dev. Neurosci..

[193] Williams J.B., Roberts S.P., Elekonich M.M. (2008). Age and natural metabolically-intensive behavior affect oxidative stress and antioxidant mechanisms. Exp. Gerontol..

[194] Schulz D.J., Barron A.B., Robinson G.E. (2002). A role for octopamine in honey bee division of labor. Brain Behav. Evolut..

[195] Higes M., Martín-Hernández R., Botías C., Bailón E.G., González-Porto A.V., Barrios L., Del Nozal M.J., Bernal J.L., Jiménez J.J., Palencia P.G. (2008). How natural infection by *Nosema ceranae* causes honeybee colony collapse. Environ. Microbiol..

[196] Hauser F., Neupert S., Williamson M., Predel R., Tanaka Y., Grimmelikhuijzen C.J.P. (2010). Genomics and Peptidomics of Neuropeptides and Protein Hormones Present in the Parasitic Wasp *Nasonia vitripennis*. J. Proteome Res..

